# The Inflammatory Response to Enterotoxigenic *E. coli* and Probiotic *E. faecium* in a Coculture Model of Porcine Intestinal Epithelial and Dendritic Cells

**DOI:** 10.1155/2018/9368295

**Published:** 2018-12-20

**Authors:** Henriette Loss, Jörg R. Aschenbach, Karsten Tedin, Friederike Ebner, Ulrike Lodemann

**Affiliations:** ^1^Institute of Veterinary Physiology, Department of Veterinary Medicine, Freie Universität Berlin, Oertzenweg 19b, 14163 Berlin, Germany; ^2^Institute of Microbiology and Epizootics, Department of Veterinary Medicine, Freie Universität Berlin, Robert-von-Ostertag-Str. 7-13, 14163 Berlin, Germany; ^3^Institute of Immunology, Department of Veterinary Medicine, Freie Universität Berlin, Robert-von-Ostertag-Str. 7-13, 14163 Berlin, Germany

## Abstract

The gut epithelium constitutes an interface between the intestinal contents and the underlying gut-associated lymphoid tissue (GALT) including dendritic cells (DC). Interactions of intestinal epithelial cells (IEC) and resident DC are characterized by bidirectional crosstalk mediated by various factors, such as transforming growth factor-*β* (TGF-*β*) and thymic stromal lymphopoietin (TSLP). In the present study, we aimed (1) to model the interplay of both cell types in a porcine *in vitro* coculture consisting of IEC (cell line IPEC-J2) and monocyte-derived DC (MoDC) and (2) to assess whether immune responses to bacteria are altered because of the interplay between IPEC-J2 cells and MoDC. With regard to the latter, we focused on the inflammasome pathway. Here, we propose caspase-13 as a promising candidate for the noncanonical inflammasome activation in pigs. We conducted challenge experiments with enterotoxigenic *Escherichia coli* (ETEC) and probiotic *Enterococcus faecium* (*E. faecium*) NCIMB 10415. As potential mediators of IEC/DC interactions, TGF-*β* and TSLP were selected for analyses. Cocultured MoDC showed attenuated ETEC-induced inflammasome-related and proinflammatory interleukin (IL)-8 reactions compared with MoDC monocultures. Caspase-13 was more strongly expressed in IPEC-J2 cells cocultured with MoDC and upon ETEC incubation. We found that IPEC-J2 cells and MoDC were capable of releasing TSLP. The latter cells secreted greater amounts of TSLP when cocultured with IPEC-J2 cells. TGF-*β* was not modulated under the present experimental conditions in either cell types. We conclude that, in the presence of IPEC-J2 cells, porcine MoDC exhibited a more tolerogenic phenotype, which might be partially regulated by autocrine TSLP production. Noncanonical inflammasome signaling appeared to be modulated in IPEC-J2 cells. Our results indicate that the reciprocal interplay of the intestinal epithelium and GALT is essential for promoting balanced immune responses.

## 1. Introduction

Intestinal epithelial cells lining the intestinal mucosa are continuously exposed to a variety of potentially harmful antigens and build a physical interface that separates the luminal content from the host milieu [1]. In the gut, DC are found in the *lamina propria*, in the subepithelial dome region of Peyer's patches, and in solitary lymph nodes such as the mesenteric lymph nodes [2–4]. Within the dynamic communication system between enterocytes and mucosal immune cells, IEC direct the function of resident DC by releasing immune mediators, such as the regulatory cytokine TGF-*β* and TSLP [5, 6]. Intestinal DC and IEC are both pivotal for maintaining normal barrier function as they support the discrimination between inflammatory and tolerogenic immune responses [7, 8]. Therefore, functional properties of the intestinal epithelium cannot be fully understood by using *in vitro* models in which epithelial cells are solely grown as monocultures [7]. Our objective was to reconstruct the intestinal environment *in vitro* by implementing the presence of MoDC in the subepithelial compartment of a porcine jejunum epithelial cell line grown on cell culture inserts of Transwell systems.

Since luminal microbiota also participate in the crosstalk [9, 10], we hypothesized that the inflammatory response patterns of IEC and immune cells to the different types of bacteria are influenced by the mutual interplay of these cells. Therefore, a pathogenic ETEC strain frequently causing postweaning diarrhea in piglets [11, 12] and an apathogenic *E. faecium* strain were included in the study design. In pigs, the probiotic *E. faecium* NCIMB 10415, which is used as a feed additive for sows and piglets, has previously been demonstrated to exert diverse favorable effects on the immune system and performance parameters both *in vitro* [13–15] and *in vivo* [16–19], especially during the postweaning period.

We aimed to unravel variations in the inflammatory responses of IEC and DC under coculture conditions with a focus on the signaling *via* the inflammasome pathway. Nucleotide oligomerization domain (NOD)-like receptors (NLR) represent a class of intracellular pattern recognition receptors (PRR), some of which are able to form inflammasomes [20]. A well-known member of this inflammasome-forming receptor family is NLRP3 (NLR family, pyrin domain containing 3) [21]. Among other stimuli, the NLRP3 inflammasome can be activated through bacterial infection [22]. Canonical and noncanonical inflammasome activations can be distinguished with regard to the characterization of inflammasome signaling [23]. Upon canonical inflammasome activation, the effector caspase-1 leads to the production and secretion of the proinflammatory cytokines IL-1*β* and IL-18 [24]. In contrast, noncanonical inflammasome activation requires species-specific inflammatory caspases other than caspase-1, particularly caspase-11 in mice [25] and caspase-4 and -5 in humans [26, 27]. Bovine caspase-13 is presumed to represent the ortholog of human caspase-4 [28]. Based on these findings, we propose that caspase-13 exerts a similar function in pigs. Noncanonical inflammasome activation has been demonstrated for various Gram-negative bacteria, such as *Vibrio cholerae*, *Escherichia coli* (*E. coli*), and *Salmonella* Typhimurium [25, 29]. Most of the inflammasome studies have been carried out in human or mouse models, but a deeper understanding of porcine inflammasome pathways is lacking. In particular, no studies exist regarding noncanonical inflammasome activation in pigs. A further hypothesis tested in the present study was that porcine caspase-13 is involved in noncanonical inflammasome activation in pigs.

## 2. Material and Methods

### 2.1. Porcine Intestinal Epithelial Cells

The cell line IPEC-J2 was used as a porcine intestinal epithelial model. The cell line was originally derived from the jejunum of a newborn piglet and was kindly provided by Professor Dr. Anthony Blikslager (North Carolina State University, USA). Cells were cultivated as described elsewhere [15]. Medium was changed 3 times per week. Every 7 days, cells were split at a ratio of 1 : 3. Passages between 73 and 80 were included in the experiments. IPEC-J2 cells were seeded on the top surface of collagenized cell culture inserts of 12-well Transwell systems (12 mm diameter, 1.12 cm^2^ growth surface area, 0.4 *μ*m pore size, Costar, Corning BV, Schiphol-Rijk, The Netherlands) at a density of 1 × 10^5^ cells per cell culture insert. Cells were cultivated under a humidified atmosphere of 5% CO_2_ at 37°C for 14 to 21 days until reaching confluency.

### 2.2. Generation of Monocyte-Derived Dendritic Cells

Blood was taken from conventionally reared Danbred × Pietrain pigs (10 to 12 weeks of age) kept at the Institute of Animal Nutrition (Freie Universität Berlin, Germany) or from clinically healthy pigs at a slaughterhouse in Brandenburg, Germany. The blood sample collection procedure was conducted in accordance with the guidelines for animal welfare and was approved by the ethics committee for animal welfare, namely, “Landesamt für Gesundheit und Soziales” (LaGeSo Berlin, no. T0264/15). Blood samples were collected in 9 ml ethylenediamine tetra-acetic acid (EDTA)-coated blood tubes (S-Monovette®, SARSTEDT, Nümbrecht, Germany).

Peripheral blood mononuclear cells (PBMC) were purified by density gradient centrifugation as described by Loss et al. [30] by using Ficoll-Paque™ PLUS (1.077 g/l, GE Healthcare, Uppsala, Sweden). Monocytes were subsequently enriched by magnetic labeling based on their CD14 expression and subsequent cell sorting in a MidiMACS separator and LS separation columns (both from Miltenyi Biotec, Bergisch Gladbach, Germany). CD14^+^ monocytes were diluted in RPMI-1640 (Biochrom, Berlin, Germany) supplemented with 10% fetal calf serum (FCS, Biochrom), 100 U/ml penicillin, and 100 *μ*g/ml streptomycin (Sigma-Aldrich Chemie GmbH). Cells were seeded at a density of 1.44 × 10^6^ cells/ml and 1 ml per well in 12-well cell culture plates (TPP, Faust Lab, Klettgau, Germany or Eppendorf GmbH, Hamburg, Germany). To differentiate monocytes into MoDC, cells were supplemented with recombinant porcine (rp) granulocyte-macrophage colony-stimulating factor (GM-CSF, 20 ng/ml) and rp IL-4 (50 ng/ml; both from R&D Systems, Minneapolis, MN, USA). Cells were grown at 37°C under a humidified atmosphere of 5% CO_2_ for 6 days. After 3 days, cells were fed with another 1 ml of fresh differentiation medium. On day 6, adherent immature MoDC were used for the experiments. In order to ensure successful differentiation, the morphological and phenotypical features of the cells were examined by phase contrast microscopy (Leica DMI 6000 series, Leica Microsystems, Heidelberg, Germany) and flow cytometry. Flow cytometric phenotypical characterization was performed as described elsewhere [30]. Briefly, the monoclonal antibodies anti-human CD14 (clone REA599, isotype IgG1, Miltenyi Biotec), anti-pig CD16 (clone G7, isotype IgG1, Bio-Rad Laboratories GmbH, Munich, Germany), anti-pig CD1 (clone 76-7-4, isotype IgG2*ακ*, SouthernBiotech, Cambridge, United Kingdom), and anti-pig swine leukocyte antigen (SLA) II (clone K274.3G8, isotype IgG1, major histocompatibility complex [MHC] II, Bio-Rad Laboratories GmbH) were used. Successful differentiation was considered to have occurred when the cells showed a characteristic DC morphology and were tested as being CD14^+^ CD16^+^ CD1^+^ SLA^+^.

### 2.3. Bacterial Strains

Two different bacterial strains were used for the experiments: the probiotic strain *E. faecium* NCIMB 10415 (Cylactin®, DSM, Kaiseraugst, Switzerland) and enterotoxigenic *E. coli* IMT4818 (isolated from a two-week-old piglet with diarrhea, O149:K91:K88 [F4]). *E. faecium* and ETEC were grown on BHI (brain-heart infusion) and LB (Luria-Bertani) agar plates, respectively. After overnight incubation, *E. faecium* was grown in BHI broth (OXOID GmbH, Wesel, Germany) and ETEC in LB medium containing 10 g/l tryptone, 5 g/l yeast extract, and 10 g/l NaCl, at pH 7.0 (Roth, Karlsruhe, Germany). Each bacterial strain was cultivated at 37°C until the midlog phase was reached. Bacteria were then centrifuged and washed twice with cold PBS. Prior to addition to IPEC-J2 cells, bacteria were diluted in serum- and antibiotic-free IPEC-J2 cell culture medium at a concentration of approximately 10^8^ colony-forming units (CFU)/ml. For their application into the MoDC compartment, RPMI-1640 was used for the resuspension of bacterial cells to give a concentration of approximately 10^7^ CFU/ml. In order to quantify bacterial concentrations, the optical density (OD) was measured at a wavelength of 600 nm in a Helios™ Epsilon spectrophotometer (Thermo Scientific, Rockford, IL). Additionally, dilution series were made with a subsequent CFU count on LB agar plates.

### 2.4. Coculture Model and Experimental Design (Figure 1)

**Figure 1:**
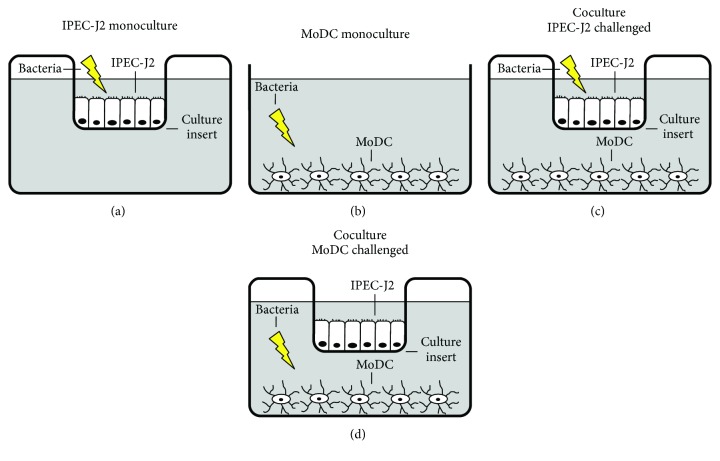
Schematic illustration of experimental design. (a) IPEC-J2 monocultures were grown as a monolayer on the top surface of Transwell cell culture inserts. Bacteria were added to the IPEC-J2 compartment. (b) MoDC monocultures were cultivated in 12-well cell culture plates. Bacteria were added to the MoDC compartment. (c)–(d) Cocultures of IPEC-J2 cells grown on Transwell inserts and adherent MoDC located in the bottom compartment. In separate approaches, bacteria were added either (c) to the IPEC-J2 compartment or (d) to the MoDC compartment. The lightning flash indicates the localization of the bacterial challenge with either *E. faecium* or ETEC.

In the present study, a coculture model comprising IPEC-J2 cells and porcine MoDC was utilized as illustrated in Figures 1(c) and 1(d). To this end, Transwell inserts with confluent IPEC-J2 monolayers grown on their top surface were transferred to the 12-well culture plates containing adherent MoDC on the bottom. On the day prior to the experiments, each cell type was fed with the appropriate cell culture medium. After 24 h in coculture, the cells were challenged with the aforementioned bacterial strains.

Prior to bacterial infection, FCS- and penicillin-streptomycin-supplemented media were removed from the cell cultures and replaced by serum- and antibiotic-free IPEC-J2 or MoDC cell culture medium, respectively, after the appropriate cells had been washed with the aforementioned media.

For the experiments, cells were incubated with either the probiotic *E. faecium* strain or the pathogenic ETEC strain. The number of bacteria differed depending on the cell type infected. IPEC-J2/MoDC cocultures were incubated with bacteria by adding either 1 × 10^6^ CFU per insert to the IPEC-J2 compartment of the cultures or 5.4 × 10^4^ CFU per well to the MoDC compartment (Figures 1(c) and 1(d)). The appropriateness of the applied bacterial concentrations was evaluated in preliminary experiments.

In addition to the IPEC-J2/MoDC cocultures, monocultures of IPEC-J2 cells or MoDC were also included as controls to assess the influence of cocultivation on the reactivity of each cell type (Figures 1(a) and 1(b)).

For the sake of completeness, we examined the immune responses after direct incubation with the bacterial strains (comparison of mono- *vs.* coculture), as well as after indirect bacterial incubation. In these assays, we additionally assessed the inflammatory responses of cocultured IPEC-J2 cells when MoDC had been challenged and *vice versa*. Thus, an *in vivo*-like situation discriminating between the apical or basolateral occurrence of individual bacteria was simulated. The expected higher bacterial load in the lumen compared with the subepithelial space was also modeled as described above; this has to be taken into account when interpreting the results of the indirect challenge.

In order to prevent bacterial overgrowth, cells were washed with gentamicin-containing medium (150 *μ*g/ml, Biochrom) after 2 h of bacterial incubation. The medium was then replaced with medium supplemented with gentamicin at a final concentration of 50 *μ*g/ml. After this medium change, cells were incubated for further 4 h.

### 2.5. Transepithelial Electrical Resistance (TEER) Measurements

The transepithelial electrical resistance (TEER) across the IPEC-J2 monolayers was measured in the Transwell systems by using a Millicell-ERS (Electrical Resistance System, Millipore GmbH, Schwalbach, Germany). During the experiments, the TEER was measured every two hours (before bacterial addition and at 2 h, 4 h, and 6 h of incubation). TEER values were corrected against their blank control (cell-free cell culture insert with medium) and against the membrane area. For each experimental condition, three wells were used. Results are reported as [*Ω* × cm^2^].

### 2.6. Real-Time Quantitative Polymerase Chain Reaction (RT-qPCR)

To perform mRNA expression analyses, samples for RT-qPCR were collected 6 h after bacterial addition. MoDC and IPEC-J2 cells were washed with cold PBS, harvested by scraping, and stored in RNA*later* RNA stabilization reagent (Qiagen GmbH, Hilden, Germany) at −20°C. Isolation of RNA and its quantitative and qualitative analyses were performed as described by Kern et al. [14]. Samples were used when the RNA integrity number was higher than or equal to 8. An aliquot of 100 ng total RNA was reverse-transcribed into cDNA in a Mastercycler™ Nexus Gradient (Eppendorf GmbH) by using the iScript™ cDNA Synthesis Kit (Bio-Rad Laboratories GmbH). All primers for RT-qPCR were synthesized by Eurofins MWG Synthesis GmbH (Ebersberg, Germany). In preliminary experiments, various reference genes were validated for each cell line by using ge-Norm® software. Three reference genes were selected for normalization (MoDC: TATA-binding protein [TBP], tyrosine 3-monooxygenase/tryptophan 5-monooxygenase activation protein zeta [YWHAZ], and beta-2-microglobulin [B2M]; IPEC-J2: TBP, YWHAZ, and glyceraldehyde-3-phosphate dehydrogenase [GAPDH]). Primer information regarding the target and reference genes is given in Table 1. RT-qPCR was conducted in an iCycler iQ™ Real-Time PCR Detection System (Bio-Rad Laboratories GmbH) by using SYBR Green I detection. Samples were run in triplicate. The final volume of the reaction (20 *μ*l) was composed of iQ SYBR Green Supermix (Bio-Rad Laboratories GmbH), primers (0.38 *μ*l of 20 pmol/*μ*l each), and 5 *μ*l cDNA. An inter-run calibration sample was used to correct for run-to-run variations. To check for possible genomic DNA contamination, minus-reverse transcriptase controls were included in the experiments. The software iQ5 (Bio-Rad Laboratories GmbH) was utilized to calculate the relative expression of target genes by using the ΔΔCt method.

### 2.7. Enzyme-Linked Immunosorbent Assay (ELISA)

For the analysis of cytokine release from MoDC or IPEC-J2 cells, cell-free cell culture supernatants were collected 6 h after bacterial addition, centrifuged (6000 rpm for 5 min), and stored at −80°C until used. IL-1*β*, IL-8, TGF-*β*, and TSLP concentrations were determined by using the following ELISA kits according to the manufacturer's instructions: porcine IL-1*β* ELISA (Quantikine ELISA, Porcine IL-1*β*/IL-1F2 Immunoassay, R&D Systems), porcine IL-8 ELISA (Invitrogen ELISA Kit, Swine IL-8, Invitrogen Life Technologies GmbH), porcine TGF-*β* ELISA (Quantikine ELISA, Porcine TGF-*β*1 Immunoassay, R&D Systems), and porcine TSLP ELISA (Porcine TSLP ELISA kit, BlueGene, Shanghai, China). A microplate reader (EnSpire Multimode Plate Reader, Perkin Elmer, Rodgau, Germany) was employed to measure the absorbance values and to calculate the OD-specific sample concentrations from a standard curve by using a four-parameter logistic curve fit. Results are reported as (pg/ml).

### 2.8. Statistical Analysis

Statistical analyses and the creation of graphs were performed by using SigmaPlot 11.0 for Windows (Systat Software Inc., San Jose, CA, USA). Statistical significance of differences between the various treatment groups was assessed by two-way repeated measures analysis of variance (ANOVA) for the factors “bacteria” (“control”, “*E. faecium*”, and “ETEC”) and “culture” (“IPEC-J2 monoculture”/“MoDC monoculture”, “coculture - IPEC-J2 challenged”, and “coculture - MoDC challenged”). In addition to the analysis of these two main effects, we also tested for possible interactions between the two factors. If interactions occurred, comparisons among the different treatment groups of the factor “bacteria” were made for each “culture” condition and *vice versa*. Findings were considered to be significant when *P* ≤ 0.05. When overall analysis of the data of each cell type and a certain parameter (TEER, mRNA, or protein expression) showed a statistical difference between treatment groups (including interactions), the Fisher least significant difference *post hoc* test was carried out. In the figures, results are presented as means ± standard error of the means (SEM).

## 3. Results

### 3.1. TEER

**Figure 2:**
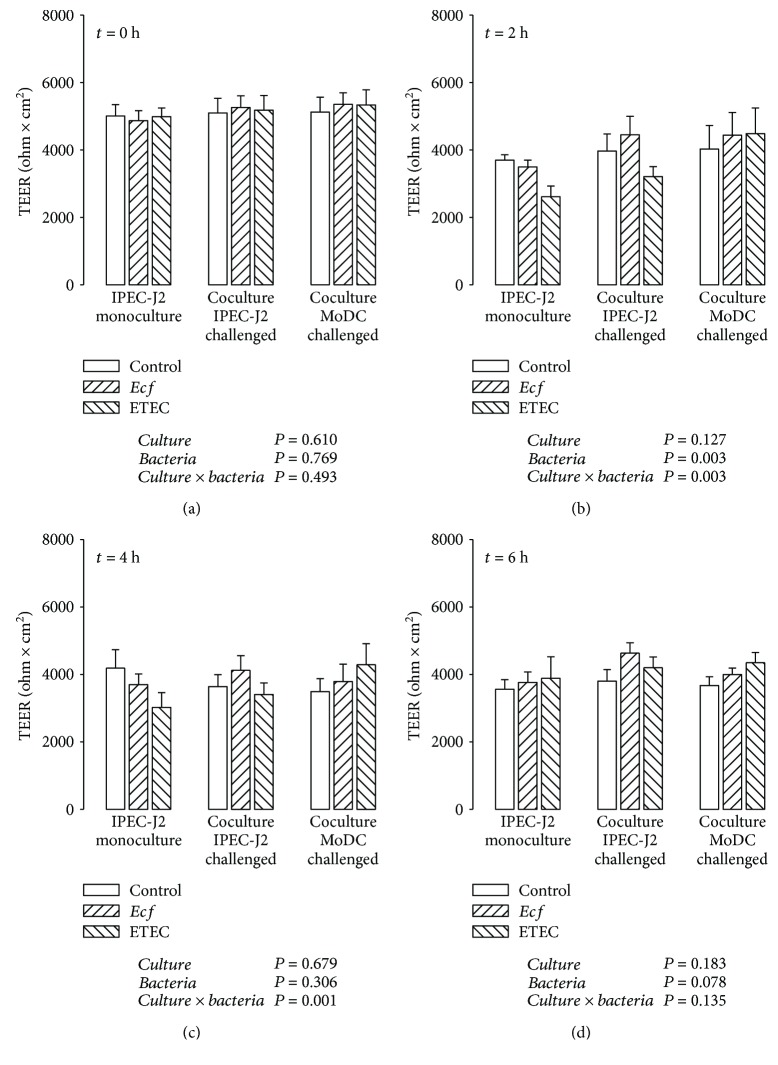
Transepithelial electrical resistance (TEER, in *Ω* × cm^2^) of IPEC-J2 monolayers after stimulation with either *E. faecium* (*Ecf*) or ETEC. In IPEC-J2/MoDC cocultures, *Ecf* or ETEC was added either to the apical side of IPEC-J2 cells or to the MoDC compartment. In IPEC-J2 monocultures, the bacteria were added to the apical compartment. TEER values were measured at 0 h, 2 h, 4 h, and 6 h (a)-(d). Data are expressed as means ± SEM. *N* = 6 independent experiments per bar. Results of the ANOVA are indicated below each graph. Results of *post hoc* tests are presented in Supplementary Table 1.

During the course of experiments, TEER values of the IPEC-J2 monolayers were measured at four time points in order to monitor the barrier integrity (Figure 2). In addition to the cocultures, TEER was also determined in corresponding IPEC-J2 monocultures. As shown in Figure 2(a), initial TEER values of the cocultures did not differ from those of IPEC-J2 monocultures before the bacterial challenge.

In IPEC-J2 monocultures, ETEC significantly reduced the TEER after 2 h (*P* ≤ 0.05) (Figure 2(b)) and after 4 h (*P* ≤ 0.05) of incubation (Figure 2(c)). In cocultures with challenged IPEC-J2 cells, this ETEC effect was only present at 2 h after bacterial infection (*P* ≤ 0.05) (Figure 2(b)). Bacterial infection of cocultured MoDC revealed no ETEC-induced drop of TEER of IPEC-J2 monolayers at each considered time point. Unlike ETEC, *E. faecium* treatment led to no modifications in the TEER throughout the experimental period in each experimental setup.

### 3.2. Expression of Inflammation-Related Genes in IPEC-J2 Cells

The mRNA expression of various inflammation-related genes was analyzed in IPEC-J2 cells (and porcine MoDC—see next section) after 6 h of bacterial stimulation. Cells were incubated with either probiotic *E. faecium* or pathogenic ETEC. Samples were obtained from cocultures or from the corresponding monocultures.

**Figure 3:**
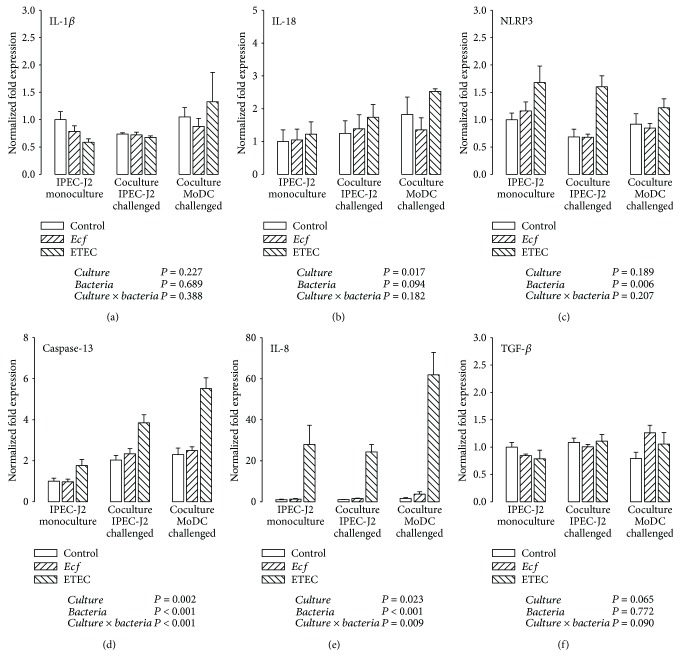
mRNA expression of (a) IL-1*β*, (b) IL-18, (c) NLRP3, (d) caspase-13, (e) IL-8, and (f) TGF-*β* in IPEC-J2 cells after stimulation with either *E. faecium* (*Ecf*) or ETEC. In IPEC-J2/MoDC cocultures, *Ecf* or ETEC was added either to the apical side of IPEC-J2 cells or to the MoDC compartment. In IPEC-J2 monocultures, the bacteria were added to the apical compartment. Samples were taken at 6 h after addition of bacteria (means ± SEM). *N* = 4 independent experiments per bar. Normalized fold expression was calculated by the ΔΔCt method. Results of the ANOVA are indicated below each graph. Results of *post hoc* tests are presented in Supplementary Table 2.

To gain insight into the potential involvement of the inflammasome pathway, IL-1*β*, IL-18, and NLRP3 were selected for the analysis of the inflammasome response to the applied bacterial strains. As shown in Figure 3(a), mRNA expression levels of IL-1*β* in IPEC-J2 cells remained rather stable independent of the cultivation method (cocultures or monocultures) and the bacterial challenge. However, the IL-18 mRNA expression of IPEC-J2 cells was generally higher in the coculture setup when MoDC had been challenged compared with IPEC-J2 monocultures (*P* ≤ 0.05) (Figure 3(b)). This effect was, as a trend, mainly based on greater values in cocultures challenged with ETEC. Incubation with the pathogenic ETEC strain also provoked an upregulation of NLRP3 mRNA expression in IPEC-J2 cells in comparison with the control and the *E. faecium* group (*P* ≤ 0.05) (Figure 3(c)).

We hypothesized that caspase-13 would be a promising candidate targeting noncanonical inflammasome activation in pigs. As indicated in Figure 3(d), caspase-13 mRNA expression was strongly enhanced in ETEC-infected IPEC-J2 cells under either cultivation methods (*P* ≤ 0.05). Notably, the observed upregulation was more evident in cocultured IPEC-J2 cells than in IPEC-J2 monocultures (*P* ≤ 0.05). An interesting additional finding was that the cocultivation of IPEC-J2 with MoDC (irrespective of infection) was followed by a higher caspase-13 mRNA expression in IPEC-J2 cells (*P* ≤ 0.05).

To further illuminate the noncanonical inflammasome signaling pathway in IPEC-J2 cells, we additionally included the following genes in our analyses: inflammasome-forming NLRC4 (NLR family, CARD domain containing 4), the adapter ASC (apoptosis-associated speck-like protein containing a CARD), caspase-1, and toll-like receptor (TLR) 4 (Supplementary Tables 6 and 7). We found a lack of NLRC4 mRNA in IPEC-J2 cells (Supplementary Table 6). Whilst ASC mRNA expression was not regulated, caspase-1 mRNA expression was upregulated in cocultured IPEC-J2 cells (*P* ≤ 0.05). TLR4 mRNA levels were higher in the ETEC-incubated treatment groups (*P* ≤ 0.05).

The mRNA expression of the proinflammatory chemokine IL-8 in IPEC-J2 cells was markedly augmented by incubation with ETEC under each culture condition (*P* ≤ 0.05) (Figure 3(e)). As with caspase-13, IPEC-J2 cells from the setting in which cocultured MoDC was treated with the bacteria exhibited the largest ETEC response (*P* ≤ 0.05).

In contrast, *E. faecium* treatment did not alter the mRNA expression of the considered genes within the experimental design and showed expression levels similar to those of the unchallenged controls.

As a regulatory cytokine, we also investigated the expression of TGF-*β*, which was affected neither by the cultivation method nor by bacterial incubation in IPEC-J2 cells (Figure 3(f)).

### 3.3. Expression of Inflammation-Related Genes in Porcine MoDC

Similarly, mRNA expression was studied in porcine MoDC. Expression levels were compared between cocultured MoDC (challenged with bacteria directly or indirectly by infection of IPEC-J2 cells) and MoDC originating from monocultures.

**Figure 4:**
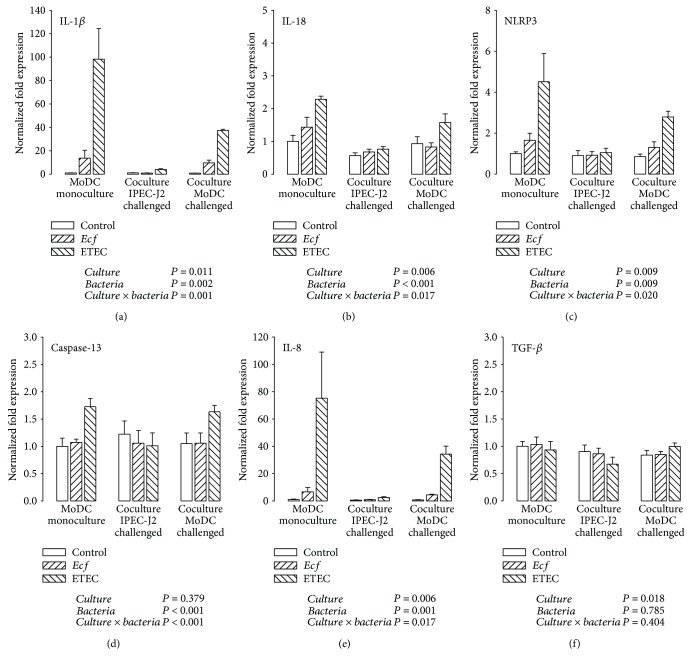
mRNA expression of (a) IL-1*β*, (b) IL-18, (c) NLRP3, (d) caspase-13, (e) IL-8, and (f) TGF-*β* in porcine MoDC after stimulation with either *E. faecium* (*Ecf*) or ETEC. In IPEC-J2/MoDC cocultures, *Ecf* or ETEC was added either to the apical side of IPEC-J2 cells or to the MoDC compartment. In MoDC monocultures, the bacteria were added to the basolateral compartment. Samples were taken at 6 h after addition of bacteria (means ± SEM). *N* = 4 independent experiments per bar. Normalized fold expression was calculated by the ΔΔCt method. Results of the ANOVA are indicated below each graph. Results of *post hoc* tests are presented in Supplementary Table 3.

The analysis of inflammasome-linked genes (IL-1*β*, IL-18, and NLRP3) revealed an upregulation by ETEC in MoDC cultivated alone (*P* ≤ 0.05) (Figures 4(a)–4(c)). To a significantly lesser extent, ETEC enhanced the mRNA expression of IL-1*β*, IL-18, and NLRP3 in the challenged MoDC of the cocultures (*P* ≤ 0.05). In addition, cocultured MoDC remained relatively unaffected by the bacterial challenge of IPEC-J2 cells. Likewise, ETEC caused an enlarged caspase-13 transcription when MoDC were challenged in mono- and cocultures (*P* ≤ 0.05) (Figure 4(d)). In contrast to genes associated with canonical inflammasome activation, the induced caspase-13 mRNA increase in cocultured MoDC was as great as in MoDC monocultures.

Expression patterns of IL-8 resembled those of IL-1*β* and NLRP3 (Figure 4(e)). The highest response to ETEC was detected in MoDC monocultures, whereas a weaker ETEC-triggered amplification of IL-8 mRNA occurred in cocultured MoDC (*P* ≤ 0.05).

Exposure to the probiotic *E. faecium* strain resulted in only a slight increase of IL-18 mRNA expression in MoDC that were cultivated alone (*P* ≤ 0.05) (Figure 4(b)). Similar tendencies were recognized for IL-1*β*, NLRP3, and IL-8 mRNA expressions without reaching statistical significance (Figures 4(a), 4(c), and 4(e)).

Similar to that of IPEC-J2 cells, the mRNA expression of anti-inflammatory TGF-*β* in MoDC showed no clear effects in the context of bacterial treatment or the cultivation technique (Figure 4(f)). On average, the smallest expression level was detected in IPEC-J2/MoDC cocultures in which IPEC-J2 cells had been bacterially challenged (*P* ≤ 0.05); this was attributable to a numerical ETEC-induced decrease.

### 3.4. Cytokine Secretion by IPEC-J2 Cells

The protein secretion of IL-8, IL-1*β*, TGF-*β*, and TSLP into cell culture supernatants of IPEC-J2 cells and porcine MoDC (see next section) was determined by ELISA. For the analysis of the selected cytokines, samples were collected 6 h after bacterial addition.

**Figure 5:**
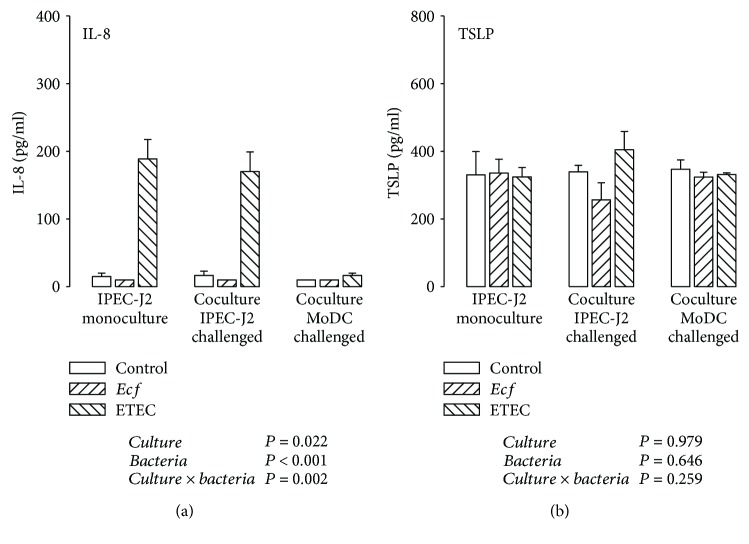
Protein expression (in pg/ml) of (a) IL-8 and (b) TSLP detected by ELISA in supernatants of IPEC-J2 cells after stimulation with either *E. faecium* (*Ecf*) or ETEC. In IPEC-J2/MoDC cocultures, *Ecf* or ETEC was added either to the apical side of IPEC-J2 cells or to the MoDC compartment. In IPEC-J2 monocultures, the bacteria were added to the apical compartment. Samples were taken at 6 h after addition of bacteria (means ± SEM). *N* = 3 independent experiments per bar. Results of the ANOVA are indicated below each graph. Results of *post hoc* tests are presented in Supplementary Table 4.

In challenged IPEC-J2 cells of mono- and cocultures, a strong secretion of IL-8 attributable to ETEC infection could be observed (*P* ≤ 0.05) (Figure 5(a)). These results corresponded with those of the qPCR analysis. Interestingly, the results after bacterial addition to the MoDC compartment varied considerably from the mRNA to the protein level. The high upregulation of IL-8 mRNA expression could not be verified at the protein level.

IPEC-J2 cells secreted TSLP, which we proposed as being a promising candidate mediating the interactive IEC/DC crosstalk in addition to TGF-*β*, at levels of around 300 pg/ml, but the detected levels did not show significant variations attributable to different cultivation variants and bacterial stimulation (Figure 5(b)).

IL-1*β* and TGF-*β* concentrations in the tested IPEC-J2 supernatant samples were mostly below the minimum detection level of the ELISA kits used (6.7 and 4.6 pg/ml, respectively; data not shown).

### 3.5. Cytokine Secretion by Porcine MoDC

**Figure 6:**
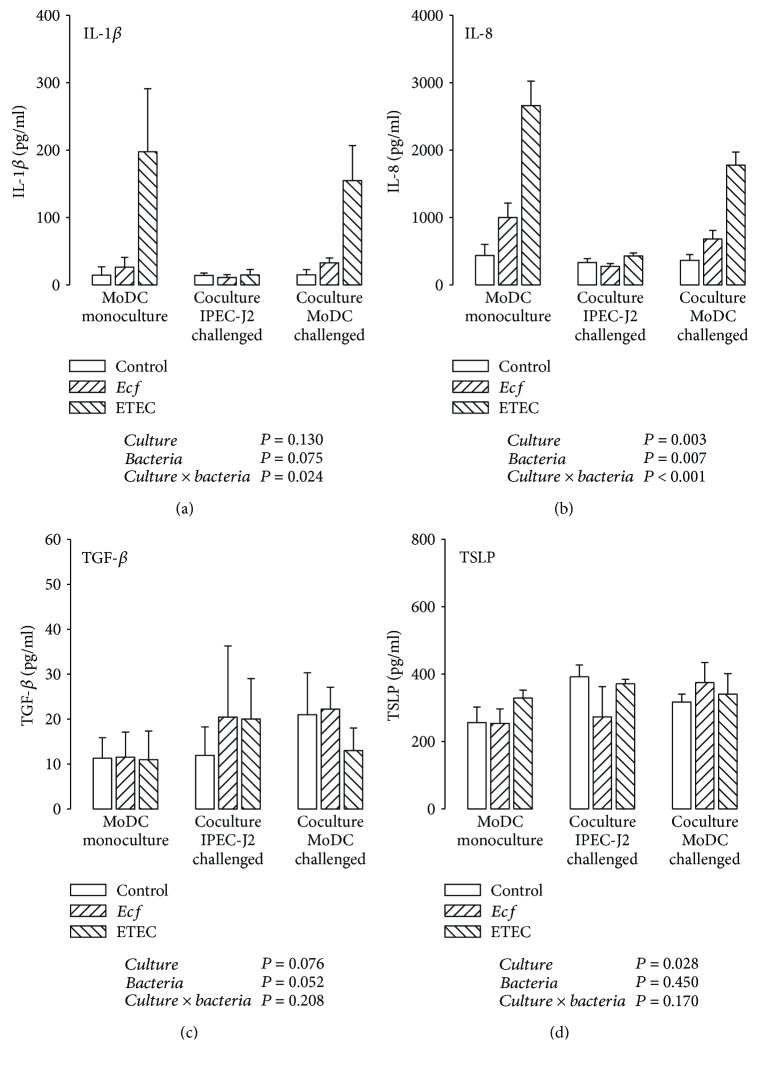
Protein expression (in pg/ml) of (a) IL-1*β*, (b) IL-8, (c) TGF-*β*, and (d) TSLP detected by ELISA in supernatants of porcine MoDC after stimulation with either *E. faecium* (*Ecf*) or ETEC. In IPEC-J2/MoDC cocultures, *Ecf* or ETEC was added either to the apical side of IPEC-J2 cells or to the MoDC compartment. In MoDC monocultures, the bacteria were added to the basolateral compartment. Samples were taken at 6 h after addition of bacteria (means ± SEM). *N* = 3 − 4 independent experiments per bar. Results of the ANOVA are indicated below each graph. Results of *post hoc* tests are presented in Supplementary Table 5.

In supernatants of mono- and cocultured MoDC, direct ETEC incubation caused an IL-1*β* accumulation (*P* ≤ 0.05) with a tendency of lower IL-1*β* concentrations in the presence of IPEC-J2 cells (Figure 6(a)).

The IL-8 release of ETEC-infected MoDC was greater in MoDC monocultures than in cocultures with IPEC-J2 cells (*P* ≤ 0.05) (Figure 6(b)). This was in agreement with results obtained at the mRNA level. Furthermore, incubation with probiotic *E. faecium* also elicited a higher IL-8 protein level in directly challenged MoDC monocultures (compared with *E. faecium* responses under the remaining culture conditions), but this was lower than the ETEC-induced increases (*P* ≤ 0.05) (Figure 6(b)).

Porcine MoDC secreted low amounts of TGF-*β* into the respective supernatants, which tended to be increased in *E. faecium*-incubated cells (*P* = 0.052) (Figure 6(c)). Surprisingly, we also detected TSLP expression at the protein level in MoDC samples (Figure 6(d)). The quantities of MoDC-derived TSLP were comparable with those measured in IPEC-J2 cells. The supernatant of MoDC cultivated in the presence of IPEC-J2 cells contained more TSLP than that of monocultures, regardless of the bacterial treatment (*P* ≤ 0.05).

## 4. Discussion

In the present study, the main objective was to determine whether the inflammatory response to a bacterial challenge in porcine MoDC and IPEC-J2 cells is changed by their mutual interference in an *in vitro* coculture model. As encountered enteric bacteria can be of different types, we conducted challenge experiments with an apathogenic *E. faecium* strain and a pathogenic *E. coli* strain, the latter having disease relevance for pigs, especially in the postweaning period.

### 4.1. TEER

The analysis of TEER values of IPEC-J2/MoDC cocultures and their corresponding IPEC-J2 monocultures revealed that the cocultivation of IPEC-J2 cells with MoDC per se did not have an effect on the TEER of IPEC-J2 monolayers. Similar findings were achieved with human intestinal models consisting of human IEC and MoDC [36, 37].

Previous studies have shown that ETEC is capable of altering the barrier function of apically infected IPEC-J2 monolayers adversely in a dose- and time-dependent manner [15, 38–40]. In the present study, ETEC effects on the barrier integrity were predominantly detectable after 2 h of incubation. Apically challenged IPEC-J2 monocultures showed a significantly lowered TEER even after 4 h, suggesting that IPEC-J2 monocultures were slightly more sensitive to ETEC-induced impairments of the epithelial barrier function than cocultures. Surprisingly, basolateral bacterial infection had no influence on TEER at any time point. The latter might be attributable to the lower number of pathogenic bacteria added to cocultured MoDC on the basolateral side of IPEC-J2 cells resulting in a lower bacteria : IPEC-J2 cell ratio. When adding the same bacterial concentration, other research groups have shown that the basolateral infection of human IEC (cell line T84) monocultures with pathogenic bacteria, such as adherent-invasive *E. coli* [41] or *Campylobacter jejuni* [42], resulted in a considerable TEER drop, which was greater after basolateral application compared with apical application. Nonetheless, the lower number of ETEC applied to the basolateral compartment of IPEC-J2 cells in the current study induced an evident proinflammatory response (see next section).

### 4.2. The Inflammatory Response in IPEC-J2 Cells

In the present study, we provide evidence that inflammasome activation following a pathogenic ETEC challenge occurred in both cell types examined. In IPEC-J2 cells, this was particularly validated by an upregulation of NLRP3 mRNA expression. In addition to the main cell wall component lipopolysaccharide (LPS), other inflammasome-stimulating components of pathogenic *E. coli* include toxins, such as enterohemolysin and heat-labile enterotoxin [43, 44], or bacterial RNA [45, 46]. The role of NLRP3 in intestinal inflammation and homeostasis is controversial [47–49]. Lissner and Siegmund [50] have underlined that the outcome depends on the affected cell type. Within the epithelium, NLRP3 performs regulatory functions, e.g., by promoting enterocyte proliferation, whereas disproportionate NLRP3 activation by *lamina propria* immune cells provokes detrimental effects [50]. A protective role of the NLRP3 inflammasome in IEC has been postulated by Song-Zhao et al. [51] and Zaki et al. [52]. In a recent study, Fan et al. [53] addressed inflammasome activation in IPEC-J2 cells upon stimulation with the mycotoxin zearalenone and reported evidence supporting regulatory functions of the NLRP3 inflammasome within the gut [53].

We further studied NLRC4 mRNA expression in IPEC-J2 cells as the NRLC4 inflammasome is another well-characterized inflammasome beyond NLRP3. We found no NLRC4 mRNA in these cells. We and others had previously reported similar findings for different porcine cells and tissues, suggesting that a functional NLRC4 gene is missing in pigs [30, 54, 55].

Whilst IEC are the main source for IL-18 being especially important for epithelial regeneration [52, 56, 57], the ability of IEC to produce IL-1*β* is a matter of debate [58]. Based on our results, IL-1*β* played a negligible role in IPEC-J2 cells, whereas IL-18 mRNA expression tended to follow similar expression patterns as determined for caspase-13, indicating that there might be a correlation between caspase-13 and IL-18.

Based on the assumption that caspase-13 is the porcine counterpart to murine caspase-11, the striking upregulation of caspase-13 mRNA expression in IPEC-J2 cells upon ETEC exposure suggests that ETEC could primarily trigger noncanonical inflammasome activation in IPEC-J2 cells. In addition, the caspase-13 induction as a result of the pathogenic ETEC challenge was more evident in cocultured IPEC-J2 cells than in IPEC-J2 monocultures. In human and murine IEC, Knodler et al. [59] have observed noncanonical inflammasome activation *via* caspase-4 and caspase-11, respectively, in response to enteropathogens. Recent research has assigned the murine ortholog caspase-11 guard functions within the gastrointestinal tract in inflammatory states [60–62]. For example, caspase-11-deficient mice revealed a hypersensitivity to dextran sulfate sodium-induced colitis associated with an impeded IL-18 production [60, 61], suggesting an ameliorating effect of caspase-11 during intestinal inflammation [63]. To date, it is unknown how inflammasome signaling by IEC is cross-linked with other defense mechanisms that ultimately coordinate the recruitment of neighboring immune cells [58].

Some authors have demonstrated that caspase-11 activation acts upstream of caspase-1-dependent canonical inflammasome formation [64, 65], whereas others have reported that caspase-11 forms a noncanonical inflammasome complex itself [25, 66]. Caspase-1, in contrast to caspase-4, -5, and -11, is capable of processing interleukins [67]. Analysis of caspase-1 mRNA expression in IPEC-J2 cells revealed an increase upon cocultivation, which had likewise been detected at the level of caspase-13 in IPEC-J2 cells, indicating a possible link between caspase-13 and caspase-1. Resembling results have been obtained in murine cocultures consisting of preadipocytes and muscle cells or fibroblasts, in which the mRNA expression of certain caspases (caspase-3, -7, and -9) was in some cases enhanced as an effect of coculturing [68, 69]. To our knowledge, similar investigations for caspases associated with noncanonical inflammasome signaling have not yet been carried out.

Furthermore, several authors have shown that the signaling pathway for caspase-11 activation includes TLR4 (and the TLR adapter TRIF [TIR domain containing adaptor inducing interferon-*β*]), which senses extracellular LPS [66, 70]. In IPEC-J2 cells, we could verify ETEC-associated upregulations of TLR4 mRNA expression, which might indicate that this signal cascade is likewise involved in porcine noncanonical inflammasome activation.

The transcriptional control differs between the caspases of different species, e.g., it has been established for murine caspase-11 but not for human caspase-4, which is constitutively expressed [71]. Summarizing the observations of the current study, it was suggested that the porcine ortholog caspase-13 responds similarly to the murine counterpart. In this respect, the verification in future studies as to whether caspase-13 constitutes the porcine equivalent to the aforementioned caspases of the noncanonical inflammasome pathway would be intriguing. Collectively, we can conclude that the DC-driven regulation of neighboring IPEC-J2 cells was mainly evidenced by caspase-13 modulation. This caspase-13 modulation might be one possible explanation for the altered TEER response observed after apical ETEC infection of IPEC-J2 mono- *vs.* cocultures and for the lack of a TEER response after basolateral ETEC infection of IPEC-J2 cells.

### 4.3. The Inflammatory Response in Porcine MoDC

Investigations into the inflammatory response in porcine MoDC revealed that MoDC from IPEC-J2/MoDC cocultures reacted more moderately to the pathogenic ETEC challenge than did monocultured MoDC; the expression of IL-1*β*, IL-18, and NLRP3 was attenuated at the mRNA level and of IL-1*β*, as a trend, also at the protein level. For the proinflammatory cytokine IL-8, a similar pattern was noted at both the mRNA and protein levels. In contrast to IEC, DC are known to express NLRP3 abundantly and to generate high IL-1*β* levels [72]. An exaggerated production of cytokines, such as IL-1*β* and IL-8, can lead to the development of intestinal pathologies linked with a disruption of the intestinal barrier, such as inflammatory bowel disease [73, 74]. Hence, our findings concerning inflammasome and IL-8 reactions support the hypothesis that IEC act beneficially to adapt the proinflammatory responsiveness of MoDC to invading enteropathogens. We propose an inflammation-restricting effect of adjacent IPEC-J2 cells on porcine MoDC in the present study. Other research groups have provided evidence that IEC are able to suppress proinflammatory responses of cocultured immune cells [37, 75]. In a human model of the intestinal epithelium, DC cultivated in direct contact with IEC were less sensitive to LPS and exhibited a reduced proinflammatory response [37].

Of note, the caspase-13 mRNA expression in MoDC did not appear to be influenced following cocultivation with IPEC-J2 cells; and the ETEC-induced caspase-13 upregulation was reduced compared with those detected in IPEC-J2 cells. We presume that the transcriptional induction of caspase-13 plays a rather minor role in porcine MoDC, at least, within our experimental design. This underlines the observation that different cell types fulfil a unique contribution to the development of immune responses, particularly in the gut [58].

The apathogenic *E. faecium* strain used in this study had only a minor impact on certain proinflammatory markers (IL-18, IL-1*β*, and IL-8) in MoDC monocultures. Comparable proinflammatory responses to different *E. faecium* strains have been documented by several working groups that recorded a strain-specific and dose-dependent induction of, for example, IL-1*β*, IL-8, IL-6, and tumor necrosis factor-*α*, in human DC or murine macrophages [76–79]. TGF-*β* was suggested to contribute to probiotic-triggered immunoregulatory mechanisms [80–82]. Accordingly, a tendency for an *E. faecium*-induced increase of TGF-*β* secretion by MoDC was also noted in our experiments.

### 4.4. Potential Mediators of Crosstalk between IPEC-J2 Cells and Porcine MoDC

As porcine MoDC revealed an attenuated inflammatory ETEC response when cocultured with IPEC-J2 cells, we aimed to look more closely at underlying IPEC-J2/MoDC interactions. In our experimental design, MoDC had no direct contact with neighboring IPEC-J2 cells. Hence, the modulation of the immune cells was assumed to occur through cell-derived humoral signals capable of crossing the filter membrane. In our analyses, we included TSLP and TGF-*β*, which we considered as potential mediators in this bidirectional crosstalk.

Consistent with the idea that soluble factors are likely to be responsible for the regulation of DC responses, Rimoldi et al. [8] demonstrated that human DC conditioned by supernatants of IEC displayed a downregulated IL-1*β* secretion after *Salmonella* infection. In their study, IEC-derived TSLP was identified as the controlling agent [8]. Although TSLP is commonly regarded as an epithelial-derived cytokine, it has previously been detected in murine [83] and human DC [84, 85], where it was released in an autocrine manner in response to pathogenic and allergenic agents. In the present study, we observed TSLP expression by both IPEC-J2 and porcine DC. Unexpectedly, MoDC-derived TSLP appeared to contribute to an autocrine regulation under coculture conditions.

An autocrine regulation mechanism of MoDC has likewise been proposed on the basis of TGF-*β* secretion [37]. Butler et al. [37] observed a higher TGF-*β* release by human DC cocultured in direct contact with IEC; this was accompanied by weaker inflammatory reactions to pathogenic stimuli, as stated earlier. However, this effect was absent in a separated coculture setup that was more similar to our IPEC-J2/MoDC cocultures [37]. According to our results, no clear impact of cocultivation on TGF-*β* expression in porcine MoDC was present, either at the mRNA or at the protein level.

Since it is unclear whether IPEC-J2 cells are capable of producing TGF-*β* [86], we measured TGF-*β* at the mRNA level. We verified TGF-*β* mRNA expression in IPEC-J2 cells but which was, however, not regulated in the different treatment groups. Consistent with our data, Butler et al. [37] detected only very small amounts of TGF-*β* liberated by IEC, so that TGF-*β* could not be identified as a modulating IEC-derived mediator in the present experimental design.

Future studies are needed to obtain knowledge as to the extent to which results may be different when a cocultivation technique is used that allows direct contact between the cocultured cell types. Here, we provide evidence supporting a possible involvement of TSLP derived by porcine MoDC in the communication between IPEC-J2 cells and MoDC.

## 5. Conclusions

In the present study, we established a porcine intestinal model consisting of IPEC-J2 cells and MoDC. We investigated inflammatory reactions to selected bacterial agents and found a more tolerogenic phenotype of MoDC cocultured with IEC. This conclusion was supported by a downregulation of inflammasome-related and other proinflammatory cytokines in comparison with MoDC cultivated alone. We further provide the first evidence that porcine caspase-13 is regulated in IPEC-J2 cells and porcine MoDC in response to bacterial infection. In IPEC-J2 cells, the possibly related noncanonical inflammasome pathway appeared to be induced not only by ETEC infection but also by the presence of MoDC. Finally, we demonstrated the ability of IPEC-J2 cells and MoDC to secrete TSLP, whereby an autocrine adaptation of cocultured MoDC was indicated. Our results suggest that the control of inflammatory responses by IEC is of critical importance to prevent unrestricted cytokine production by resident immune cells. More research is needed to unravel further the soluble factors that are implicated in IEC/DC interactions and to verify the functional aspects of porcine caspase-13 in noncanonical inflammasome signaling. We suggest the presented *in vitro* coculture model is a promising tool for studying such interactions in future.

## Figures and Tables

**Table 1:** Oligonucleotide primers and amplicon length of PCR products.

Gene information	Primer sequence	Amplicon length	Accession number	Reference
*IL1B1* (interleukin-1, beta 1, *Sus scrofa*)	(S) 5′- CCT CCT CCC AGG CCT TCT GT -3′ (AS) 5′- GGG CCA GCC AGCA CTA GAG A -3′	178 bp		[31]
*IL-18* (interleukin-18, *Sus scrofa*)	(S) 5′- ACG ATG AAG ACC TGG AAT CG -3′ (AS) 5′- GCC AGA CCT CTA GTG AGG CTA -3′	205 bp	AF191088.1	
*NLRP3* (NLR family, pyrin domain containing 3, *Sus scrofa*)	(S) 5′- AGC AGA TTC CAG TGC ATC AAA G -3′ (AS) 5′- CCT GGT GAA GCG TTT GTT GAG-3′	75 bp	NM_001256770.2	[32]
*NLRC4* (NLR family, CARD domain containing 4, *Sus scrofa*)	(S) 5′- TGC TCT GAA ACA CCT TGC AT -3′ (AS) 5′- GCA TAG ATT CCT GCC TCC AG -3′	92 bp	XM_013987922.1	
*CASP13* (caspase-13, apoptosis-related cysteine peptidase, *Sus scrofa*)	(S) 5′- GTG CTA CAG AAA CGC CAT GA -3′ (AS) 5′- AGG GCA AAG CTT GAG GGT AT-3′	150 bp	XM_003129812.6	
*CASP1* (caspase-1, apoptosis-related cysteine peptidase, *Sus scrofa*)	(S) 5′- CTC TCC ACA GGT TCA CAA TC -3′ (AS) 5′- GAA GAC GCA GGC TTA ACT GG -3′	116 bp	NM_214162	[33]
*ASC (LOC100522011)* (apoptosis-associated speck-like protein containing a CARD, *Sus scrofa*)	(S) 5′- CCG ACG AGC TCA AGA AGT TT -3′ (AS) 5′- AGC TCA GCG CTG TAC TCC TC -3′	154 bp	XM_003124468.4	
*IL-8* (interleukin-8, *Sus scrofa*)	(S) 5′- GGC AGT TTT CCT GCT TTC T -3′ (AS) 5′- CAG TGG GGT CCA CTC TCA AT -3′	154 bp	X61151	[34]
*TLR4* (toll-like receptor 4, *Sus scrofa*)	(S) 5′- AGA ACT GCA GGT GCT GGA TT -3′ (AS) 5′- AGG TTT GTC TCA ACG GCA AC -3′	180 bp	AB188301	
*TGF-β* (transforming growth factor beta, *Sus scrofa*)	(S) 5′- TGA CCC GCA GAG AGG CTA TA -3′ (AS) 5′- CAT GAG GAG CAG GAA GGG C -3′	164 bp	NM_214015.2	
*TBP* (TATA box binding protein, *Sus scrofa*)	(S) 5′- GAT GGA CGT TCG GTT TAG G -3′ (AS) 5′- AGC AGC ACA GTA CGA GCA A -3′	124 bp	DQ178129	[35]
*YWHAZ* (tyrosine 3-monooxygenase/tryptophan 5-monooxygenase activation protein, zeta polypeptide, *Sus scrofa*)	(S) 5′- ATG CAA CCA ACA CAT CCT ATC -3′ (AS) 5′- GCA TTA TTA GCG TGC TGT CTT -3′	178 bp	DQ178130	[35]
*B2M* (beta-2-microglobulin, *Sus scrofa*)	(S) 5′- AAA CGG AAA GCC AAA TTA CC -3′ (AS) 5′- ATC CAC AGC GTT AGG AGT GA -3′	178 bp	DQ178123	[35]
*GAPDH* (glyceraldehyde-3-phosphate dehydrogenase, *Sus scrofa)*	(S) 5′- ACT CAC TCT TCT ACC TTT GAT GCT -3′ (AS) 5′- TGT TGC TGT AGC CAA ATT CA -3′	100 bp	DQ178124	[35]

## Data Availability

The TEER, ELISA, and qPCR data used to support the findings of this study are included within the supplementary information file.

## Abbreviations

ANOVA: Analysis of variance
ASC: Apoptosis-associated speck-like protein containing a CARD
BHI: Brain-heart infusion
B2M: Beta-2-microglobulin
CFU: Colony-forming unit
DC: Dendritic cells
*E. coli*: *Escherichia coli*
EDTA: Ethylenediamine tetra-acetic acid
*E. faecium*: *Enterococcus faecium*
ELISA: Enzyme-linked immunosorbent assay
ETEC: Enterotoxigenic *Escherichia coli*
FCS: Fetal calf serum
GALT: Gut-associated lymphoid tissue
GAPDH: Glyceraldehyde-3-phosphate dehydrogenase
GM-CSF: Granulocyte-macrophage colony-stimulating factor
IEC: Intestinal epithelial cells
IL: Interleukin
LB: Luria-Bertani
LPS: Lipopolysaccharide
MHC: Major histocompatibility complex
MoDC: Monocyte-derived DC
NLR: NOD-like receptor
NLRC4: NLR family, CARD domain containing 4
NLRP3: NLR family, pyrin domain containing 3
NOD: Nucleotide oligomerization domain
OD: Optical density
PBMC: Peripheral blood mononuclear cells
PBS: Phosphate-buffered saline
PRR: Pattern recognition receptors
rp: Recombinant porcine
RT-qPCR: Real-time quantitative polymerase chain reaction
SEM: Standard error of the means
SLA: Swine leukocyte antigen
TBP: TATA-binding protein
TEER: Transepithelial electrical resistance
TGF-*β*: Transforming growth factor-*β*
TLR: Toll-like receptor
TSLP: Thymic stromal lymphopoietin
YWHAZ: Tyrosine 3-monooxygenase/tryptophan 5-monooxygenase activation protein zeta.

## Supplementary Materials

Supplementary Materials In Supplementary Table 1, the results of the post hoc tests of the TEER data are presented. Supplementary Tables 2, 3, 6, and 7 show the results of the post hoc tests of the mRNA expression analyses in IPEC-J2 cells and MoDC. In the Supplementary Tables 4 and 5, those results are presented for the protein expression analyses in IPEC-J2 cells and MoDC.
