# Supplementary material for: The Inflammatory Response to Enterotoxigenic E. coli and Probiotic E. faecium in a Coculture Model of Porcine Intestinal Epithelial and Dendritic Cells
Source: Mediators Inflamm. 2018 Dec 20;2018:9368295. doi: 10.1155/2018/9368295 (PMC6317115; doi:10.1155/2018/9368295)
Supplement: Supplementary Materials — In Supplementary Table 1, the results of the post hoc tests of the TEER data are presented. Supplementary Tables 2, 3, 6, and 7 show the results of the post hoc tests of the mRNA expression analyses in IPEC-J2 cells and MoDC. In the Supplementary Tables 4 and 5, those results are presented for the protein expression analyses in IPEC-J2 cells and MoDC. [file 9368295.f1.docx]

## Supplementary materials

Supplementary table 1: Transepithelial electrical resistance (TEER, in Ω×cm^2^) of IPEC-J2 monolayers after stimulation with either *E. faecium* (*Ecf*) or ETEC.

In IPEC-J2/MoDC co-cultures, *Ecf* or ETEC were added either to the apical side of IPEC-J2 cells or to the MoDC compartment. In IPEC-J2 monocultures, the bacteria were added to the apical compartment. TEER values were measured at 0 h, 2 h, 4 h, and 6 h (a)-(d). Data are expressed as least square means (LSM) ± SEM. N = 6 independent experiments. Different lower case letters indicate significant differences between treatment groups within each column for the factor bacteria (*P* ≤ 0.05) and different capital letters within each row for the factor culture (*P* ≤ 0.05).

| **(a) t = 0 h** | **IPEC-J2 monoculture** | **Co-culture –**  **IPEC-J2 challenged** | **Co-culture –**  **MoDC challenged** | **LSM ± SEM bacteria** | ***P* value bacteria** |
| --- | --- | --- | --- | --- | --- |
| **Control** | 5005±109 | 5096±109 | 5122±109 | 5074 ±97 | *P* = 0.769 |
| ***E. faecium*** | 4865 ±109 | 5258±109 | 5352±109 | 5159±97 |  |
| **ETEC** | 4985±109 | 5174±109 | 5331±109 | 5164±97 |  |
| **LSM ± SEM culture** | 4952±226 | 5176±226 | 5268±226 |  | Bacteria × culture |
| ***P* value culture** | *P* = 0.610 |  |  |  | *P* = 0.493 |

| **(b) t = 2 h** | **IPEC-J2 monoculture** | **Co-culture –**  **IPEC-J2 challenged** | **Co-culture –**  **MoDC challenged** | **LSM ± SEM bacteria** | ***P* value bacteria** |
| --- | --- | --- | --- | --- | --- |
| **Control** | 3694±187^a^ | 3965±187^a^ | 4024±187 | 3894±104 | *P* = 0.003 |
| ***E. faecium*** | 3496±187^a^ | 4447±187^a^ | 4435 ±187 | 4126±104 |  |
| **ETEC** | 2612±187^b,B^ | 3209±187^b,B^ | 4482±187^A^ | 3434±104 |  |
| **LSM ± SEM culture** | 3267±329 | 3874±329 | 4314±329 |  | Bacteria × culture |
| ***P* value culture** | *P* = 0.127 |  |  |  | *P* = 0.003 |

| **(c) t = 4 h** | **IPEC-J2 monoculture** | **Co-culture –**  **IPEC-J2 challenged** | **Co-culture –**  **MoDC challenged** | **LSM ± SEM bacteria** | ***P* value bacteria** |
| --- | --- | --- | --- | --- | --- |
| **Control** | 4184±204^a^ | 3635±204^ab^ | 3486 ±204^b^ | 3769±132 | *P* = 0.306 |
| ***E. faecium*** | 3694±204^a^ | 4123±204^a^ | 3789±204^ab^ | 3869±132 |  |
| **ETEC** | 3019±204^b,B^ | 3402±204^b,B^ | 4284±204^a,A^ | 3568±132 |  |
| **LSM ± SEM culture** | 3632±175 | 3720 ±175 | 3853±175 |  | Bacteria × culture |
| ***P* value culture** | *P* = 0.679 |  |  |  | *P* = 0.001 |

| **(d) t = 6 h** | **IPEC-J2 monoculture** | **Co-culture –**  **IPEC-J2 challenged** | **Co-culture –**  **MoDC challenged** | **LSM ± SEM bacteria** | ***P* value bacteria** |
| --- | --- | --- | --- | --- | --- |
| **Control** | 3560±161 | 3799±161 | 3667±161 | 3675±146 | *P* = 0.078 |
| ***E. faecium*** | 3761±161 | 4631±161 | 3995±161 | 4129±146 |  |
| **ETEC** | 3883±161 | 4198±161 | 4346±161 | 4142±146 |  |
| **LSM ± SEM culture** | 3735±167 | 4209±167 | 4002±167 |  | Bacteria × culture |
| ***P* value culture** | *P* = 0.183 |  |  |  | *P* = 0.135 |

Supplementary table 2: mRNA expression of (a) IL-1β, (b) IL-18, (c) NLRP3, (d) caspase-13, (e) IL-8, and (f) TGF-β in IPEC-J2 cells after stimulation with either *E. faecium* (*Ecf*) or ETEC.

In IPEC-J2/MoDC co-cultures, *Ecf* or ETEC were added either to the apical side of IPEC-J2 cells or to the MoDC compartment. In IPEC-J2 monocultures, the bacteria were added to the apical compartment. Samples were taken at 6 h after addition of bacteria [least square means ± SEM]. N = 4 independent experiments. Normalized fold expression was calculated by the ΔΔCt method. Different lower case letters indicate significant differences between treatment groups within each column for the factor bacteria (*P* ≤ 0.05) and different capital letters within each row for the factor culture (*P* ≤ 0.05).

| **(a) IL-1β** | **IPEC-J2 monoculture** | **Co-culture –**  **IPEC-J2 challenged** | **Co-culture –**  **MoDC challenged** | **LSM ± SEM bacteria** | ***P* value bacteria** |
| --- | --- | --- | --- | --- | --- |
| **Control** | 1.00±0.19 | 0.74±0.19 | 1.05±0.19 | 0.93±0.11 | *P* = 0.689 |
| ***E. faecium*** | 0.78±0.19 | 0.72±0.19 | 0.87±0.19 | 0.79±0.11 |  |
| **ETEC** | 0.59±0.19 | 0.68±0.19 | 1.33±0.19 | 0.86±0.11 |  |
| **LSM ± SEM culture** | 0.79±0.14 | 0.71±0.14 | 1.08±0.14 |  | Bacteria × culture |
| ***P* value culture** | *P* = 0.227 |  |  |  | *P* = 0.388 |

| **(b) IL-18** | **IPEC-J2 monoculture** | **Co-culture –**  **IPEC-J2 challenged** | **Co-culture –**  **MoDC challenged** | **LSM ± SEM bacteria** | ***P* value bacteria** |
| --- | --- | --- | --- | --- | --- |
| **Control** | 1.00±0.20 | 1.25±0.20 | 1.82±0.20 | 1.36±0.16 | *P* = 0.094 |
| ***E. faecium*** | 1.05±0.20 | 1.39±0.20 | 1.36±0.20 | 1.26±0.16 |  |
| **ETEC** | 1.20±0.20 | 1.74±0.20 | 2.53±0.20 | 1.82±0.16 |  |
| **LSM ± SEM culture** | 1.08±0.14^B^ | 1.46±0.14^AB^ | 1.90±0.14^A^ |  | Bacteria × culture |
| ***P* value culture** | *P* = 0.017 |  |  |  | *P* = 0.182 |

| **(c) NLRP3** | **IPEC-J2 monoculture** | **Co-culture –**  **IPEC-J2 challenged** | **Co-culture –**  **MoDC challenged** | **LSM ± SEM bacteria** | ***P* value bacteria** |
| --- | --- | --- | --- | --- | --- |
| **Control** | 1.00±0.14 | 0.68±0.14 | 0.92±0.14 | 0.87±0.10^b^ | *P* = 0.006 |
| ***E. faecium*** | 1.16±0.14 | 0.68±0.14 | 0.85±0.14 | 0.90±0.10^b^ |  |
| **ETEC** | 1.68±0.14 | 1.60±0.14 | 1.22±0.14 | 1.50±0.10^a^ |  |
| **LSM ± SEM culture** | 1.28±0.11 | 0.99±0.11 | 0.99±0.11 |  | Bacteria × culture |
| ***P* value culture** | *P* = 0.189 |  |  |  | *P* = 0.207 |

| **(d) Caspase-13** | **IPEC-J2 monoculture** | **Co-culture –**  **IPEC-J2 challenged** | **Co-culture –**  **MoDC challenged** | **LSM ± SEM bacteria** | ***P* value bacteria** |
| --- | --- | --- | --- | --- | --- |
| **Control** | 1.00±0.20^b,B^ | 2.03±0.20^b,A^ | 2.30±0.20^b,A^ | 1.78±0.14 | *P* < 0.001 |
| ***E. faecium*** | 0.97±0.20^b,B^ | 2.33±0.20^b,A^ | 2.49±0.20^b,A^ | 1.93±0.14 |  |
| **ETEC** | 1.76±0.20^a,C^ | 3.85±0.20^a,B^ | 5.52±0.20^a,A^ | 3.71±0.14 |  |
| **LSM ± SEM culture** | 1.24±0.25 | 2.74±0.25 | 3.43±0.25 |  | Bacteria × culture |
| ***P* value culture** | *P* = 0.002 |  |  |  | *P* < 0.001 |

| **(e) IL-8** | **IPEC-J2 monoculture** | **Co-culture –**  **IPEC-J2 challenged** | **Co-culture –**  **MoDC challenged** | **LSM ± SEM bacteria** | ***P* value bacteria** |
| --- | --- | --- | --- | --- | --- |
| **Control** | 1.00±4.86^b^ | 1.00±4.86^b^ | 1.62±4.86^b^ | 1.21±3.03 | *P* < 0.001 |
| ***E. faecium*** | 1.28±4.86^b^ | 1.58±4.86^b^ | 3.73±4.86^b^ | 2.20±3.03 |  |
| **ETEC** | 27.90±4.86^a,B^ | 24.29±4.86^a,B^ | 61.88±4.86^a,A^ | 38.02±3.03 |  |
| **LSM ± SEM culture** | 10.06±2.71 | 8.96±2.71 | 22.41±2.71 |  | Bacteria × culture |
| ***P* value culture** | *P* = 0.023 |  |  |  | *P* = 0.009 |

| **(f) TGF-β** | **IPEC-J2 monoculture** | **Co-culture –**  **IPEC-J2 challenged** | **Co-culture –**  **MoDC challenged** | **LSM ± SEM bacteria** | ***P* value bacteria** |
| --- | --- | --- | --- | --- | --- |
| **Control** | 1.00±0.11 | 1.08±0.11 | 0.79±0.11 | 0.96±0.08 | *P* = 0.772 |
| ***E. faecium*** | 0.84±0.11 | 1.00±0.11 | 1.26±0.11 | 1.04±0.08 |  |
| **ETEC** | 0.79±0.11 | 1.11±0.11 | 1.06±0.11 | 0.98±0.08 |  |
| **LSM ± SEM culture** | 0.88±0.05 | 1.07±0.05 | 1.04±0.05 |  | Bacteria × culture |
| ***P* value culture** | *P* = 0.065 |  |  |  | *P* = 0.090 |

Supplementary table 3: mRNA expression of (a) IL-1β, (b) IL-18, (c) NLRP3, (d) caspase-13, (e) IL-8, and (f) TGF-β in porcine MoDC after stimulation with either *E. faecium* (*Ecf*) or ETEC.

In IPEC-J2/MoDC co-cultures, *Ecf* or ETEC were added either to the apical side of IPEC-J2 cells or to the MoDC compartment. In MoDC monocultures, the bacteria were added to the basolateral compartment. Samples were taken at 6 h after addition of bacteria [least square means ± SEM]. N = 4 independent experiments. Normalized fold expression was calculated by the ΔΔCt method. Different lower case letters indicate significant differences between treatment groups within each column for the factor bacteria (*P* ≤ 0.05) and different capital letters within each row for the factor culture (*P* ≤ 0.05).

| **(a) IL-1β** | **MoDC monoculture** | **Co-culture –**  **IPEC-J2 challenged** | **Co-culture –**  **MoDC challenged** | **LSM ± SEM bacteria** | ***P* value bacteria** |
| --- | --- | --- | --- | --- | --- |
| **Control** | 1.00±8.55^b^ | 1.11±8.55 | 0.89±8.55^b^ | 1.00±5.21 | *P* = 0.002 |
| ***E. faecium*** | 13.68±8.55^b^ | 0.89±8.55 | 9.62±8.55^b^ | 8.06±5.21 |  |
| **ETEC** | 98.22±8.55^a,A^ | 3.95±8.55^C^ | 37.29±8.55^a,B^ | 46.49±5.21 |  |
| **LSM ± SEM culture** | 37.63±5.57 | 1.98±5.57 | 15.93±5.57 |  | Bacteria × culture |
| ***P* value culture** | *P* = 0.011 |  |  |  | *P* = 0.001 |

| **(b) IL-18** | **MoDC monoculture** | **Co-culture –**  **IPEC-J2 challenged** | **Co-culture –**  **MoDC challenged** | **LSM ± SEM bacteria** | ***P* value bacteria** |
| --- | --- | --- | --- | --- | --- |
| **Control** | 1.00±0.14^c^ | 0.57±0.14 | 0.93±0.14^b^ | 0.83±0.07 | *P* < 0.001 |
| ***E. faecium*** | 1.43±0.14^b,A^ | 0.68±0.14^B^ | 0.83±0.14^b,B^ | 0.98±0.07 |  |
| **ETEC** | 2.29±0.14^a,A^ | 0.76±0.14^C^ | 1.58±0.14^a,B^ | 1.54±0.07 |  |
| **LSM ± SEM culture** | 1.57±0.12 | 0.67±0.12 | 1.11±0.12 |  | Bacteria × culture |
| ***P* value culture** | *P* = 0.006 |  |  |  | *P* = 0.017 |

| **(c) NLRP3** | **MoDC monoculture** | **Co-culture –**  **IPEC-J2 challenged** | **Co-culture –**  **MoDC challenged** | **LSM ± SEM bacteria** | ***P* value bacteria** |
| --- | --- | --- | --- | --- | --- |
| **Control** | 1.00±0.43^b^ | 0.91±0.43 | 0.86±0.43^b^ | 0.92±0.30 | *P* = 0.009 |
| ***E. faecium*** | 1.65±0.43^b^ | 0.92±0.43 | 1.30±0.43^b^ | 1.29±0.30 |  |
| **ETEC** | 4.51±0.43^a,A^ | 1.05±0.43^C^ | 2.79±0.43^a,B^ | 2.79±0.30 |  |
| **LSM ± SEM culture** | 2.39±0.21 | 0.96±0.21 | 1.65±0.21 |  | Bacteria × culture |
| ***P* value culture** | *P* = 0.009 |  |  |  | *P* = 0.020 |

| **(d) Caspase-13** | **MoDC monoculture** | **Co-culture –**  **IPEC-J2 challenged** | **Co-culture –**  **MoDC challenged** | **LSM ± SEM bacteria** | ***P* value bacteria** |
| --- | --- | --- | --- | --- | --- |
| **Control** | 1.00±0.07^b^ | 1.22±0.07^a^ | 1.05±0.07^b^ | 1.09±0.03 | *P* < 0.001 |
| ***E. faecium*** | 1.07±0.07^b^ | 1.06±0.07^b^ | 1.06±0.07^b^ | 1.06±0.03 |  |
| **ETEC** | 1.73±0.07^a,A^ | 1.01±0.07^b,B^ | 1.63±0.07^a,A^ | 1.46±0.03 |  |
| **LSM ± SEM culture** | 1.27±0.09 | 1.10±0.09 | 1.25±0.09 |  | Bacteria × culture |
| ***P* value culture** | *P* = 0.379 |  |  |  | *P* < 0.001 |

| **(e) IL-8** | **MoDC monoculture** | **Co-culture –**  **IPEC-J2 challenged** | **Co-culture –**  **MoDC challenged** | **LSM ± SEM bacteria** | ***P* value bacteria** |
| --- | --- | --- | --- | --- | --- |
| **Control** | 1.00±10.60^b^ | 0.57±10.60 | 0.68±10.60 | 0.75±7.39 | *P* = 0.023 |
| ***E. faecium*** | 6.64±10.60^b^ | 0.89±10.60 | 4.40±10.60 | 3.98±7.39 |  |
| **ETEC** | 75.20±10.60^a,A^ | 2.45±10.60^C^ | 34.27±10.60^B^ | 37.31±7.39 |  |
| **LSM ± SEM culture** | 27.61±6.29 | 1.30±6.29 | 13.11±6.29 |  | Bacteria × culture |
| ***P* value culture** | *P* = 0.067 |  |  |  | *P* = 0.036 |

| **(f) TGF-β** | **MoDC monoculture** | **Co-culture –**  **IPEC-J2 challenged** | **Co-culture –**  **MoDC challenged** | **LSM ± SEM bacteria** | ***P* value bacteria** |
| --- | --- | --- | --- | --- | --- |
| **Control** | 1.00±0.10 | 0.90±0.10 | 0.84±0.10 | 0.91±0.05 | *P* = 0.785 |
| ***E. faecium*** | 1.03±0.10 | 0.86±0.10 | 0.85±0.10 | 0.91±0.05 |  |
| **ETEC** | 0.93±0.10 | 0.67±0.10 | 1.00±0.10 | 0.87±0.05 |  |
| **LSM ± SEM culture** | 0.99±0.03^A^ | 0.81±0.03^B^ | 0.89±0.03^AB^ |  | Bacteria × culture |
| ***P* value culture** | *P* = 0.018 |  |  |  | *P* = 0.404 |

Supplementary table 4: Protein expression (in pg/mL) of (a) IL-8 and (b) TSLP detected by ELISA in supernatants of IPEC-J2 cells after stimulation with either *E. faecium* (*Ecf*) or ETEC.

In IPEC-J2/MoDC co-cultures, *Ecf* or ETEC were added either to the apical side of IPEC-J2 cells or to the MoDC compartment. In IPEC-J2 monocultures, the bacteria were added to the apical compartment. Samples were taken at 6 h after addition of bacteria [least square means ± SEM]. N = 3 independent experiments. Different lower case letters indicate significant differences between treatment groups within each column for the factor bacteria (*P* ≤ 0.05) and different capital letters within each row for the factor culture (*P* ≤ 0.05).

| **(a) IL-8** | **IPEC-J2 monoculture** | **Co-culture –**  **IPEC-J2 challenged** | **Co-culture –**  **MoDC challenged** | **LSM ± SEM bacteria** | ***P* value bacteria** |
| --- | --- | --- | --- | --- | --- |
| **Control** | 15.0±16.2^b^ | 16.4±16.2^b^ | 10.0±16.2 | 13.8±3.1 | *P* < 0.001 |
| ***E. faecium*** | 10.0±16.2^b^ | 10.0±16.2^b^ | 10.0±16.2 | 10.0±3.1 |  |
| **ETEC** | 188.6±16.2^a,A^ | 169.9±16.2^a,A^ | 16.6±16.2^B^ | 125.0±3.1 |  |
| **LSM ± SEM culture** | 71.2±9.6 | 65.4±9.6 | 12.2±9.6 |  | Bacteria × culture |
| ***P* value culture** | *P* = 0.022 |  |  |  | *P* = 0.002 |

| **(b) TSLP** | **IPEC-J2 monoculture** | **Co-culture –**  **IPEC-J2 challenged** | **Co-culture –**  **MoDC challenged** | **LSM ± SEM bacteria** | ***P* value bacteria** |
| --- | --- | --- | --- | --- | --- |
| **Control** | 330±34 | 339±34 | 347±34 | 339±35 | *P* = 0.646 |
| ***E. faecium*** | 335±34 | 256±34 | 324±34 | 305±35 |  |
| **ETEC** | 324±34 | 404±34 | 331±34 | 353±35 |  |
| **LSM ± SEM culture** | 330±15 | 333±15 | 334±15 |  | Bacteria × culture |
| ***P* value culture** | *P* = 0.979 |  |  |  | *P* = 0.259 |

Supplementary table 5: Protein expression (in pg/mL) of (a) IL-1β, (b) IL-8, (c) TGF-β, and (d) TSLP detected by ELISA in supernatants of porcine MoDC after stimulation with either *E. faecium* (*Ecf*) or ETEC.

In IPEC-J2/MoDC co-cultures, *Ecf* or ETEC were added either to the apical side of IPEC-J2 cells or to the MoDC compartment. In MoDC monocultures, the bacteria were added to the basolateral compartment. Samples were taken at 6 h after addition of bacteria [least square means ± SEM]. N = 3-4 independent experiments. Different lower case letters indicate significant differences between treatment groups within each column for the factor bacteria (*P* ≤ 0.05) and different capital letters within each row for the factor culture (*P* ≤ 0.05).

| **(a) IL-1β** | **MoDC monoculture** | **Co-culture –**  **IPEC-J2 challenged** | **Co-culture –**  **MoDC challenged** | **LSM ± SEM bacteria** | ***P* value bacteria** |
| --- | --- | --- | --- | --- | --- |
| **Control** | 14.4±23.2^b^ | 14.0±23.2 | 14.9±23.2^b^ | 14.4±26.0 | *P* = 0.075 |
| ***E. faecium*** | 26.3±23.2^b^ | 10.7±23.2 | 32.8±23.2^b^ | 23.2±26.0 |  |
| **ETEC** | 197.5±23.2^a,A^ | 14.7±23.2^B^ | 154.6±23.2^a,A^ | 122.3±26.0 |  |
| **LSM ± SEM culture** | 79.4±18.8 | 13.1±18.8 | 67.5±18.8 |  | Bacteria × culture |
| ***P* value culture** | *P* = 0.130 |  |  |  | *P* = 0.024 |

| **(b) IL-8** | **MoDC monoculture** | **Co-culture –**  **IPEC-J2 challenged** | **Co-culture –**  **MoDC challenged** | **LSM ± SEM bacteria** | ***P* value bacteria** |
| --- | --- | --- | --- | --- | --- |
| **Control** | 436±136^b^ | 331±136 | 364±136^b^ | 377±137 | *P* = 0.007 |
| ***E. faecium*** | 999±136^b,A^ | 275±136^B^ | 681±136^b,AB^ | 652±137 |  |
| **ETEC** | 2660±136^a,A^ | 429±136^C^ | 1775±136^a,B^ | 1621±137 |  |
| **LSM ± SEM culture** | 1365±91 | 345±91 | 940±91 |  | Bacteria × culture |
| ***P* value culture** | *P* = 0.003 |  |  |  | *P* < 0.001 |

| **(c) TGF-β** | **MoDC monoculture** | **Co-culture –**  **IPEC-J2 challenged** | **Co-culture –**  **MoDC challenged** | **LSM ± SEM bacteria** | ***P* value bacteria** |
| --- | --- | --- | --- | --- | --- |
| **Control** | 11.3±3.2 | 12.0±3.2 | 21.0±3.2 | 14.7±0.8 | *P* = 0.052 |
| ***E. faecium*** | 11.5±3.2 | 20.3±4.3 | 22.2±3.2 | 18.0±0.9 |  |
| **ETEC** | 11.0±3.2 | 20.0±3.2 | 13.0±3.2 | 14.7±0.8 |  |
| **LSM ± SEM culture** | 11.3±2.0 | 17.4±2.2 | 18.7±2.0 |  | Bacteria × culture |
| ***P* value culture** | *P* = 0.076 |  |  |  | *P* = 0.208 |

| **(d) TSLP** | **MoDC monoculture** | **Co-culture –**  **IPEC-J2 challenged** | **Co-culture –**  **MoDC challenged** | **LSM ± SEM bacteria** | ***P* value bacteria** |
| --- | --- | --- | --- | --- | --- |
| **Control** | 256±35 | 392±35 | 317±35 | 322±23 | *P* = 0.450 |
| ***E. faecium*** | 254±35 | 273±35 | 375±35 | 300±23 |  |
| **ETEC** | 329±35 | 371±35 | 341±35 | 347±23 |  |
| **LSM ± SEM culture** | 279±12^B^ | 345±12^A^ | 344±12^A^ |  | Bacteria × culture |
| ***P* value culture** | *P* = 0.028 |  |  |  | *P* = 0.170 |

Supplementary table 6: Results of RT-qPCR for NLRC4 mRNA expression in IPEC-J2 cells. Samples were taken at 6 h after addition of bacteria. N = 4 independent experiments.

| **Sample/negative control** | **Culture** | **Bacteria** | **Mean Ct** | **Ct SD** | **% of water** |
| --- | --- | --- | --- | --- | --- |
| Sample | IPEC-J2 monoculture | Control | 33.7 | 0.3 | 101.2 |
| Sample | IPEC-J2 monoculture | *E. faecium* | 33.1 | 0.3 | 103.0 |
| Sample | IPEC-J2 monoculture | ETEC | 32.9 | 0.3 | 103.6 |
| Sample | Co-culture – IPEC-J2 challenged | Control | 31.9 | 0.2 | 106.9 |
| Sample | Co-culture – IPEC-J2 challenged | *E. faecium* | 32.2 | 0.1 | 105.9 |
| Sample | Co-culture – IPEC-J2 challenged | ETEC | 31.6 | 0.2 | 107.9 |
| Sample | Co-culture – MoDC challenged | Control | 33.5 | 0.5 | 101.8 |
| Sample | Co-culture – MoDC challenged | *E. faecium* | 32.2 | 0.5 | 105.9 |
| Sample | Co-culture – MoDC challenged | ETEC | 32.2 | 0.3 | 105.9 |
| Sample | IPEC-J2 monoculture | Control | 33.3 | 0.1 | 102.4 |
| Sample | IPEC-J2 monoculture | *E. faecium* | 32.7 | 0.2 | 104.3 |
| Sample | IPEC-J2 monoculture | ETEC | 33.6 | 0.3 | 101.5 |
| Sample | Co-culture – IPEC-J2 challenged | Control | 32.3 | 0.6 | 105.6 |
| Sample | Co-culture – IPEC-J2 challenged | *E. faecium* | 32.1 | 0.4 | 106.2 |
| Sample | Co-culture – IPEC-J2 challenged | ETEC | 32.5 | 0.4 | 104.9 |
| Sample | Co-culture – MoDC challenged | Control | 32.4 | 0.4 | 105.2 |
| Sample | Co-culture – MoDC challenged | *E. faecium* | 32.1 | 0.1 | 106.2 |
| Sample | Co-culture – MoDC challenged | ETEC | 31.9 | 0.4 | 119.1 |
| Sample | IPEC-J2 monoculture | Control | 32.8 | 0.1 | 104.0 |
| Sample | IPEC-J2 monoculture | *E. faecium* | 33.9 | 0.4 | 100.6 |
| Sample | IPEC-J2 monoculture | ETEC | 33.2 | 0.5 | 102.7 |
| Sample | Co-culture – IPEC-J2 challenged | Control | 31.3 | 0.7 | 108.9 |
| Sample | Co-culture – IPEC-J2 challenged | *E. faecium* | 32.0 | 0.3 | 106.6 |
| Sample | Co-culture – IPEC-J2 challenged | ETEC | 32.0 | 0.4 | 106.6 |
| Sample | Co-culture – MoDC challenged | Control | 32.4 | 0.2 | 105.2 |
| Sample | Co-culture – MoDC challenged | *E. faecium* | 31.9 | 0.6 | 106.9 |
| Sample | Co-culture – MoDC challenged | ETEC | 31.5 | 0.3 | 108.3 |
| Sample | IPEC-J2 monoculture | Control | 33.4 | 0.6 | 102.1 |
| Sample | IPEC-J2 monoculture | *E. faecium* | 32.8 | 0.2 | 104.0 |
| Sample | IPEC-J2 monoculture | ETEC | 34.3 | 0.3 | 99.4 |
| Sample | Co-culture – IPEC-J2 challenged | Control | 32.7 | 0.2 | 101.2 |
| Sample | Co-culture – IPEC-J2 challenged | *E. faecium* | 32.6 | 0.8 | 104.6 |
| Sample | Co-culture – IPEC-J2 challenged | ETEC | 32.3 | 0.2 | 105.6 |
| Sample | Co-culture – MoDC challenged | Control | 32.3 | 0.8 | 105.6 |
| Sample | Co-culture – MoDC challenged | *E. faecium* | 32.9 | 0.3 | 103.6 |
| Sample | Co-culture – MoDC challenged | ETEC | 32.9 | 0.1 | 103.6 |
| Negative control | (Water) | (Water) | 34.1 | 0.8 | 100.0 |

Supplementary table 7: mRNA expression of (a) ASC, (b) caspase-1, and (c) TLR4 in IPEC-J2 cells after stimulation with either *E. faecium* (*Ecf*) or ETEC.

In IPEC-J2/MoDC co-cultures, *Ecf* or ETEC were added either to the apical side of IPEC-J2 cells or to the MoDC compartment. In IPEC-J2 monocultures, the bacteria were added to the apical compartment. Samples were taken at 6 h after addition of bacteria [least square means ± SEM]. N = 4 independent experiments. Normalized fold expression was calculated by the ΔΔCt method. Different lower case letters indicate significant differences between treatment groups within each column for the factor bacteria (*P* ≤ 0.05) and different capital letters within each row for the factor culture (*P* ≤ 0.05).

| **(a) ASC** | **IPEC-J2 monoculture** | **Co-culture –**  **IPEC-J2 challenged** | **Co-culture –**  **MoDC challenged** | **LSM ± SEM bacteria** | ***P* value bacteria** |
| --- | --- | --- | --- | --- | --- |
| **Control** | 1.00±0.20 | 1.00±0.20 | 0.94±0.20 | 0.98±0.09 | *P* = 0.200 |
| ***E. faecium*** | 1.06±0.20 | 0.82±0.20 | 0.81±0.20 | 0.90±0.09 |  |
| **ETEC** | 1.30±0.20 | 1.15±0.20 | 0.99±0.20 | 1.15±0.09 |  |
| **LSM ± SEM culture** | 1.12±0.07 | 0.99±0.07 | 0.91±0.07 |  | Bacteria × culture |
| ***P* value culture** | *P* = 0.179 |  |  |  | *P* = 0.954 |

| **(b) Caspase-1** | **IPEC-J2 monoculture** | **Co-culture –**  **IPEC-J2 challenged** | **Co-culture –**  **MoDC challenged** | **LSM ± SEM bacteria** | ***P* value bacteria** |
| --- | --- | --- | --- | --- | --- |
| **Control** | 1.00±0.11 | 1.52±0.11 | 1.72±0.11 | 1.41±0.06 | *P* = 0.363 |
| ***E. faecium*** | 0.98±0.11 | 1.55±0.11 | 1.51±0.11 | 1.35±0.06 |  |
| **ETEC** | 0.88±0.11 | 1.66±0.11 | 1.88±0.11 | 1.47±0.06 |  |
| **LSM ± SEM culture** | 0.95±0.13^B^ | 1.58±0.13^A^ | 1.70±0.13^A^ |  | Bacteria × culture |
| ***P* value culture** | *P* = 0.014 |  |  |  | *P* = 0.298 |

| **(c) TLR4** | **IPEC-J2 monoculture** | **Co-culture –**  **IPEC-J2 challenged** | **Co-culture –**  **MoDC challenged** | **LSM ± SEM bacteria** | ***P* value bacteria** |
| --- | --- | --- | --- | --- | --- |
| **Control** | 1.00±0.07 | 0.84±0.07 | 0.99±0.07 | 0.94±0.08^b^ | *P* = 0.042 |
| ***E. faecium*** | 1.03±0.07 | 0.87±0.07 | 1.04±0.07 | 0.98±0.08^b^ |  |
| **ETEC** | 1.25±0.07 | 1.17±0.07 | 1.41±0.07 | 1.28±0.08^a^ |  |
| **LSM ± SEM culture** | 1.09±0.12 | 0.96±0.12 | 1.15±0.12 |  | Bacteria × culture |
| ***P* value culture** | *P* = 0.532 |  |  |  | *P* = 0.755 |
